# Promoting Resilience in Early Childhood Education and Care to Prepare Children for a World of Change: A Critical Analysis of National and International Policy Documents

**DOI:** 10.3390/children10040716

**Published:** 2023-04-12

**Authors:** Ann-Christin Furu, Angel Chan, Jonna Larsson, Ingrid Engdahl, Sarah Klaus, Anna May Navarrete, Barbara Turk Niskač

**Affiliations:** 1 Faculty of Education and Welfare Studies, Åbo Akademi University, 65101 Vasa, Finland; 2 Faculty of Education and Social Work, University of Auckland, Auckland 1142, New Zealand; 3 Department of Education, Communication and Learning, University of Gothenburg, 40530 Gothenburg, Sweden; 4 School of Education, Culture, and Communication, Mälardalen University, 72123 Västerås, Sweden; 5 Center for Child and Human Development, Georgetown University, Washington, DC 20007, USA; 6 Faculty of Education and Culture, University of Tampere, 33014 Tampere, Finland

**Keywords:** early childhood education and care, resilience, sustainability, critical document analysis

## Abstract

In recent years, the sustainability crisis has raised interest in the concept of resilience, i.e., the capacity to persist, adapt, or transform in the face of change and challenge. However, to date, resilience has only been studied to a limited extent within early childhood education and care (ECEC). This paper reports on a study that used critical document analysis of national and international policies to explore if and how the concept of resilience within ECEC could contribute to sustainability in a world of rapid change. Five national and four international documents were analysed through the theoretical lenses of childism and place-based education. The results show that resilience is implicitly expressed in ECEC policies yet is rarely linked to sustainability issues. Instead, policies mainly limit resilience to the psychological dimension and the individual child. The conclusion is that ECEC is an apt context for supporting resilience in multiple ways. It suggests using a holistic understanding of resilience to advocate for ECEC policies that include diverse perspectives of families and local communities, incorporate indigenous voices, and recognise the interconnectedness between humans and the more-than-human world.

## 1. Introduction

The intensity of the sustainability crisis is becoming apparent in research, education, and in the everyday lives of billions of people around the world. The magnitude of climate change, loss of biodiversity, pollution of air, water, and land, as well as the impact of human land use highlight the urgency of transforming our ways of living as a species. We are facing a ‘planetary emergency’ that has unprecedented impacts not only on human health and well-being, but also on the health and well-being of the multiple planetary systems on which we all rely. At the moment, five of the nine planetary boundaries—the evidence-based thresholds set for critical features of planet Earth that are essential for human stability, including climate change—have been transcended [1] and we are risking irreversible changes. Our civilisation needs to change. Individuals, communities, companies, and societies must adapt to new circumstances and mitigate further disruption. The situation is especially challenging for children across the globe [2], and education plays a crucial role in protecting their basic right to live and flourish. Early childhood education and care (ECEC) holds the potential for strengthening children’s capacity to handle challenges and contribute to change, which makes it a vibrant arena for the transformation towards sustainability [3].

There is international agreement on the importance of ECEC in this process. The *Tashkent Declaration and Commitments to Action for Transforming Early Childhood Care and Education* [4] defines ECEC as encompassing the period up to age 8. The declaration recognises the holistic nature of child development, including emotional, cognitive, and social development. ECEC entails foundational learning, responsive care, nutrition, health and well-being, safety, protection, and play. Further, it contributes to an individual’s well-being, capacity to learn, school readiness, academic achievement, lifelong learning, meaningful employment and, within society, greater gender equality, sustainable development, and global citizenship. Another example is the *Nurturing Care for Early Childhood Development* [5], which highlights the pivotal role of early learning for the youngest children in supporting individual health, well-being, and development as well as the sustainability of societies. Importantly, it approaches young children’s health and well-being as a multi-generational, cross-sectoral endeavour that links the well-being of children to their caregivers and the communities in which they reside.

These two international documents illustrate the critical role of ECEC in supporting children’s holistic learning and development, including a growing knowledge of how to care for planet Earth and a sense of responsibility in sustainability. However, resilience is essential for individuals, including children and their families, to respond to ongoing challenges and adversities presented by the climate crisis, bounce back, and persist in caring for the well-being of all inhabitants. Resilience, now a widely used concept within society, media, and research, is a vital capacity for meeting the sustainability crisis. Resilience implies the capacity to handle challenges, changes, adversity, or crisis in a flexible way [6,7,8]. We argue, in line with Elliott, Ärlemalm-Hagsér and Davis [9], that there is a need to promote a deeper understanding of how ECEC and especially early childhood education for sustainability (ECEfS) can support the transformation towards sustainability.

To our knowledge, there are few studies linking resilience to ECEfS. This article explores to what extent resilience is expressed in national and international policy documents and how ECEC might contribute to resilience in a world of change. By focusing on if and how resilience is addressed in the national policies of different countries, the article fills a gap concerning the role of resilience in ECEC. The analysis includes ECEC national policies from Finland, New Zealand, the Philippines, Slovenia, and Sweden and four recent international documents. It contributes to a deeper understanding of how ECEC can strengthen the capacities that empower children and communities to handle change and the crises they encounter.

### 1.1. Contemporary Perspectives on Resilience

Resilience implies not only coping or persisting with change, but also adapting and transforming to survive and flourish when the circumstances are challenging [10]. Ungar [7] describes how the term resilience has emerged across disciplines and is applied to systems such as ecosystems, communities, or structures in society. According to contemporary research by Millican and Middleton [11], resilience is understood as a relational capacity developed in interpersonal relationships. Hence, it is not a trait or disposition owned solely by the individual but also a characteristic shared by a group or community. It is built and strengthened, both in relationships between humans as well as in relationships between humans and nature. Further, resilience is a dynamic phenomenon, which can be perceived as stronger or weaker depending on the context. In recent years, there has been a shift from focusing on risk and adversity of the individual towards resources and strengths within the context in which the child lives, foregrounding the importance of empowerment and agency as well as the community aspects of resilience [5,6].

In previous ECEC research, resilience has been understood as a capacity of the individual child and the focus has been on its psychological dimension [12,13,14,15,16,17]. Furu and Heikkilä [18] present a relational and holistic theoretical perspective on resilience in the ECEC context, including three interrelated physical, psychological, and social dimensions. Physical resilience is related to bodily capacities to meet challenges. In our daily lives, it is strengthened by meeting the basic needs of sleep, movement, nutrition, and relaxation. In the context of ECEC, one could argue that play, care, and learning are basic needs. During an extreme event such as war or natural disaster, physical resilience can also be the capacity to find safety, shelter, food, and clothing. Psychological resilience is related to the mental and emotional capacities needed to handle change or adversity. This includes self-regulation, problem-solving, and skills related to a flexible mindset and positive feelings such as hope. Social resilience is the capacity to develop social and communicative competence and interact with other people in a meaningful and constructive manner. It entails capacities such as creating networks and collaborating to solve problems and conflicts. Further, social resilience is linked to a sense of belonging to a group or community and being capable of contributing to its well-being.

Children develop resilience within supportive and nurturing relationships in which they can build a sense of mastery and develop executive function and self-regulation as well as in contexts that affirm faith or cultural traditions [19]. ECEC plays an important part in this process [10]. Characteristics such as a sense of belonging, positive communication, and opportunities to learn new skills are key factors that support the development of resilience. Studies show that contact and connectedness with nature build psychological resilience during childhood and support pro-environmental behaviours [13,20,21]. Eriksen ∅degaard [22] argues that ECEfS pedagogy should be explorative, collaborative, child-centered, and inclusive to strengthen children’s resilience. Thus, strengthening the resilience of all children is an important aspect of ECEfS to meet the intentions of both the *Sustainable Development Goals* [23] and the *Convention on the Rights of the Child* [24]. Resilience is important not only for children growing up under disadvantaged circumstances but for all children.

### 1.2. Theoretical Framework

We conducted our study within a relational ontological framework [25] implying that development, learning, and transformation are relational processes that occur during interactions. Furthermore, we viewed humans as deeply interconnected with the more-than-human world [26]. More specifically, the study was underpinned by two distinctive but related theoretical perspectives: childism [27,28] and place-based education [29,30,31]. Childism was regarded as relevant as it emphasises listening to children´s voices and considering children as agentic. Place-based education is a valuable perspective in that it sees children as interconnected with other humans as well as with the more-than-human world.

#### 1.2.1. Childism

Wall´s [27,28] theory about childism emanates from childhood studies as a cross-subject approach. Early studies of childhood within sociology argued that children are a distinct social group with special rights and that they should be studied on this basis [32]. Studies within psychology and pedagogy further developed the field of childhood psychology in which the individual child was described as an actor with competences as opposed to the pre-dominant view of the needy, vulnerable child [33].

The concept of childism is a term that embraces not only a transforming view on children and childhood but also emphasises equality of life. Childism aims to empower the third of humanity who are under eighteen. It is analogous to other critical terms, such as feminism, in that it responds to young people’s marginalised experiences by transforming scholarly, social, and political norms and structures. It provides a critical lens for deconstructing adultism and reconstructing age-inclusive practices. Childism, therefore, recognises children as central participants in society and as rights-holders with valid voices and an ability to use them.

As Wall [27] states: “Childism focuses on transforming understandings and practices, not just around children themselves, or even around child–adult inter-generationality, but also around the pervasive normative assumptions that ground scholarship and societies overall” [27]. In our understanding, acknowledging children as rights-holders is about valuing and listening to children’s voices and, as adults, acknowledging children’s evolving status. Shining a light on freedom of speech, participation, and agency is also imperative for securing and establishing children as citizens, although this is perhaps often taken for granted. Education for and with children is historically dominated by an interest in their adaptation to the rules of an adult-centred world, rather than to children’s own possibilities. Biswas [34] illustrates how these new understandings might define the very nature of what it means to do something with children. This article adopts a ‘childist’ [28] theoretical positioning and seeks to challenge the dominance of adult social norms.

#### 1.2.2. Place-Based Education

This paper also drew on key ideas from a critical pedagogy of place approach [29,30,31] to analyse how policies in ECEC promote the notion of resilience. Gruenewald [31] states that “places teach us who, what, and where we are, as well as how we might live our lives”. According to Gruenewald [29], places are socially, culturally, and politically constructed, and place and community are synonymous in place-based education.

When Gruenewald [29] justifies the value of place-based education, he explains that “our cultural experience is ‘placed’ in the ‘geography’ of our everyday lives, and in the ‘ecology’ of the diverse relationships that take place within and between places” [29], and he suggests using culturally responsive practices to embrace geographical diversity. He further argues that “from an ecological perspective, people, cultures, and places are inescapably interconnected in relational systems” [29] because while cultural and geographical places shape humans, humans also shape the places they inhabit, thereby illustrating reciprocal and intertwining ecological relationships. Ecological relationships and interdependence, whether human–human or human–more-than-human, and within and between communities, are central to understanding the places in which we live and how we use social actions to improve these places for future inhabitants in the years, decades, and centuries to come. The politics of difference and identity, which involve the inclusion and exclusion of certain communities, also play a crucial role in interrogating relationships and power relations.

Critical practitioners challenge existing policies and the status quo to address social inequalities [29]. However, Gruenwald [29] warns that policymakers often consume words such as social justice and inequality as rhetorical and unproblematic slogans without first engaging, analysing, and understanding the context (that is, the place) in which these policies are turned into practice. Gruenewald [30], therefore, suggests combining critical pedagogy and place-based education, that is, a critical pedagogy of place, to evaluate and transform educational policies and practices. Articulating a critical pedagogy of place is thus a reaction to educational policies and practices that disregard places, overemphasise economic development, and ignore the role of relationships in education. According to Gruenewald [29,30], a pedagogical approach requires the twin processes of cultural decolonisation and ecological rehabilitation. It aims to empower people to transform and improve the ecology of places. The process of decolonisation first identifies and recognises human culture-constructed damage to places and unlearns the habituated damaging actions. Thereafter, rehabilitation undoes this damage, repairs human–human and human–more-than-human relationships, and relearns “how to live well together in a place” [29] without doing damage to others. A critical pedagogy of place, therefore, has the potential to recover relationships and interdependence and contribute to building a sustainable future world.

## 2. Materials and Methods

The study was conducted as a critical document analysis [35] of national and international policy documents in ECEC. In the following, we first describe the research materials and then the process of analysis.

### 2.1. Research Materials

The following national policy documents from Finland, New Zealand, the Philippines, Slovenia, and Sweden were included in the study. They cover children aged between 0 and 5/6 years, depending on the structure of each national educational system:

**Finland**

*National Core Curriculum for Early Childhood Education and Care* [36]
*National Core Curriculum for Pre-Primary* [37]

The *National Core Curriculum for Early Childhood Education and Care* is directed towards settings that work with children aged 0 to 5 years. The *National Core Curriculum for Pre-Primary* concerns education for children aged 6 years. The central aims of ECEC are to promote the holistic growth, development, health, and well-being of every child according to the child’s age and development and to guide the child towards ethically responsible and sustainable action, respect of other people, and membership of society.

**New Zealand**

*Te Whāriki: He Whāriki Mātauranga mō ngā Mokopuna o Aotearoa/Early Childhood Curriculum* [38]
*He Māpuna te Tamaiti: Supporting Social and Emotional Competence in Early Learning* [39]

*Te Whāriki* is the mandatory early childhood curriculum for children from birth to age five when they usually start primary school. *He Māpuna te Tamaiti* is a national resource underpinned by “key pedagogical approaches promoted in *Te Whāriki*” [39]. It aims to support EC Kaiako (teachers) “to understand and draw on effective practices that enhance children’s social and emotional competence, engagement, and learning” [39].

**The Philippines**

*Philippine Kindergarten Curriculum Guide for five-year-olds* [40]
*National Early Learning Curriculum* [41]

The Philippines has two steering documents that cover early childhood programs from 0–5 years. *The National Early Learning Curriculum* is targeted towards children aged 0–4 [41], and the *Philippine Kindergarten Guide for five-year-olds* [40], which is the first step in the Philippines’ basic education system.

**Slovenia**

*National Curriculum for Preschools* [42]

The Slovenian curriculum focuses on play, creative activities, and active learning. It emphasises the role of teachers in providing an encouraging environment in which children can learn at their own pace and have the space to *express* themselves. The curriculum divides its content among two age groups: one to three years old and three to six years old, and into six interconnected areas: movement, language, art, society, nature, and mathematics.

**Sweden**

*Curriculum for the Preschool: Lpfö 18* [43]

The *Curriculum for the preschool: Lpfö 18* covers the ages 1–5 years. It is a goal-oriented document that states the task and fundamental values, such as laying the foundation for a growing interest and responsibility among children for active participation in civic life and for sustainable development. Children have the right to participation where the needs and interests expressed in different ways by the children themselves should provide the foundation for shaping the environment and planning education.

Each country mentioned above has its own approaches and traditions in ECEC. Their policy documents were written between 1999 and 2022, which could potentially reflect different views on the urgency of and approach to sustainability issues. Together, these documents provide a holistic perspective of ECEC in both Asia and Europe. Four international policy documents were included in the analysis:

*Tashkent Declaration and Commitments to Action for Transforming Early Childhood Care and Education [Tashkent Declaration]* [8]. This document was adopted at the latest UN high-level meeting of member states on ECEC. It is thus the latest UN policy on ECEC and relates to SD Goal No 4 ensuring education for all.

*A future for the world´s children? A WHO-UNICEF-Lancet Commission [WHO-UNICEF-Lancet Commission]* [2]. This publication was produced by the Lancet Commission, which brought together editors from *The Lancet* and academic partners to identify the most pressing issues in science, medicine, and global health (in this case, the future of the world’s children) in order to provide recommendations for changing global health policy or improving practice.

*Most vulnerable to most valuable: A scoping study to put young children at the heart of climate actions and environmental protection [ARNEC document]* [44]. This study consolidated a global impact survey and desk review of global documents, sub-regional analyses, and case studies from the Asia-Pacific region, drawing together recommendations to inform effective policies and practices that promote a child-centred approach to climate action and environmental protection.

*European Union Council Recommendation on High Quality Early Childhood Education and Care [EUCR document]* [45]. This document was the result of a process within the European Union, including member states and a group of experts in the field, with the task of proposing a quality framework for ECEC in the EU.

These four documents represent the most recent international agreements highlighting the importance of ECEC in promoting sustainable societies. They express core values regarding children, childhood, and ECEC agreed upon by the global community and put forth aims and methods to support the health, well-being, development, and education of children. Further, they give a picture of how quality ECEC is constructed in policy and how ECEC can contribute to flourishing communities. The two regional documents provide guidelines for addressing climate/environmental issues (ARNEC) and enhancing quality in ECEC (EUCR). Their regional focus aligns with the analysed national policy documents.

### 2.2. The Process of Analysis

Content relevant to the research question was systematically summarised, analysed, and interpreted to identify explicit and implicit messages on resilience. The process was conducted in three steps, which involved all of the authors. The first step was a descriptive analysis of how resilience is or is not expressed in the five national policies, recent international declarations, and reports. As resilience was not always explicitly mentioned, the analysis involved identifying the following three clusters of concepts based upon the current theoretical understanding of the concept of resilience in the context of ECEC:

- agency, actor, citizenship, identity, participation
- health, well-being, holistic, integration
- connectedness, belonging, relationship, inclusion

Paragraphs and sections containing these concepts were extracted and compared between the documents.

The second step involved identifying patterns across national documents and looking into what is expressed (or not) at the following levels: child, family, community, culture, and environment. This step was conducted in online meetings with all authors.

Finally, connections and contradictions between national and international discourses were interpreted using the theoretical perspectives of childism and place-based education to gain a broader picture of how resilience is or is not expressed and how it can be supported in ECEC with respect to the mounting sustainability crisis.

## 3. Results

The findings from the study respond to the research questions: to what extent is resilience expressed in national and international policy documents and how might ECEC contribute to resilience in a world of change? The results are presented as three themes representing the versatile relationships that support resilience.

### 3.1. Relationships within the Child and between Children

All of the national policy documents present a view of the child as a capable, interconnected, agentic citizen with inherent rights. They express notions of ECEC building capacities that can be linked to all three dimensions of resilience. Supporting children´s physical resilience is mainly expressed as teaching children to take care of their health and well-being. Two patterns emerged: one being that physical resilience is related to a context of extreme weather and natural disasters, and the other being that physical resilience is related to everyday life. In the Philippines’ curriculum, the importance of physical health and well-being is related to guiding children towards developing readiness in the face of natural disasters. Standards pertaining to understanding the physical and natural environment are also indicated in the curriculum, which involve building children’s ability to care for the environment. The Finnish curricula describe the aim of ECEC as being to guide children towards ways of living that promote health and well-being from a perspective linked to everyday life, presumably under stable conditions. This highlights the importance of children learning to manage their physical resilience through actively engaging in indoor and outdoor activities. Children’s well-being is promoted by providing them with an opportunity to rest during the day as well as versatile, healthy nutrition. All curricula acknowledge the importance of movement and play in promoting feelings of joy, security, satisfaction, self-confidence, self-esteem, and intellectual skills.

Strengthening children´s psychological resilience is apparent in all national policy documents, described as nurturing their emotional capacities such as self-worth and self-esteem as well as supporting the development of cognitive capacities such as self-regulation and perseverance. For example, underpinned by the New Zealand curriculum, in *He Māpuna te Tamaiti* [39], “helping children build resilience and a sense of self-worth” [39] is identified as a strategy to support well-being. The resource explains that “confident children with good self-esteem are more resilient and able to manage their emotions” [39]. Further, it states that it is important “to understand and draw on effective practices that enhance children’s social and emotional competence, engagement, and learning” [39]. The documents of New Zealand also mention the importance of caring relationships. In the Slovenian curriculum, children´s artistic expressions are understood as important building blocks for children´s self-worth and self-esteem [42].

Building children´s social resilience is expressed as fostering a disposition of care and respect both for themselves and others. A common pattern in all documents is a strong emphasis on providing conditions in which children can develop a sense of security and social belonging. This is based on values such as equality and non-discrimination. Overall, in the five national policies analysed, children’s rights are emphasised along with notions of agency and independence, and children are encouraged to be active decision-makers, positioning them as contributors to the wider society. In the Slovenian curriculum, children should have many opportunities to develop critical minds, personal decision-making, and autonomous judgment. Most clearly, the link to ECEfS is shown in the Swedish curriculum: “The preschool should provide each child with the conditions to develop a growing responsibility for and interest in sustainable development and active participation in society” [43].

All documents consider the child as a playful learner who applies curiosity and joyful exploration to achieve the objectives set by the curriculum. The emphasis is on enabling children to express their inner selves and develop their individuality, while interacting with both humans and the natural world. This reveals the importance of creating space for play in ECEC to strengthen resilience among children.

Thus, at the level of the child, resilience is predominantly implicitly expressed. Further, resilience is understood as an individual capacity supported within a respectful, playful learning community that views the child as resourceful and capable of developing attitudes, knowledge, and skills that will enable them to handle change and challenges. From an ECECfS perspective, it is notable that explicit notions of natural disasters and undesirable events are only present in the Philippines’ curriculum.

The international documents are to some extent in line with the national documents in their focus on building resilience by supporting social and emotional skills and children’s agency. The *WHO-UNICEF-Lancet Commission* places children at the heart of the endeavour to create a sustainable world. Although resilience is not explicitly mentioned, there are multiple expressions that refer to supporting children´s well-being, health, and agency as a way of enabling them to handle and mitigate the sustainability crisis. The *Tashkent Declaration* states that ECEC should support children in developing the skills, values, and attitudes needed to tackle current and future global challenges such as climate change, biodiversity loss, health pandemics, and the erosion of democratic values and human rights. It mentions resilience once: “All [ECEC] programmes should help children and their families to build resilience to navigate future crises” [8].

The *ARNEC document* repeatedly links resilience with young children and sustainability, emphasising children’s agency. It proposes that policy development must acknowledge children as both individuals with rights and as agents of change within the community. It advocates for including discussions of climate change and environmental degradation in ECEC in order to increase resilience as well as mitigate the future impacts of climate change and environmental degradation. It envisions a role for children as ‘citizen scientists’ [44], and knowledgeable participants in policy development. The *EUCR document*, on the other hand, does not explicitly mention resilience but takes a strong stance on children’s participation: “early childhood education and care services need to be child-centered, actively involve children and acknowledge children’s views” [45].

### 3.2. Relationship between the Child, Family, and Community

Several patterns can be identified relating resilience to families, ECEC settings, and the community. In national documents, ECEC is seen as a means of preparing children so that they can be (a) part of resilient communities, and (b) contributors to build resilient communities. The *EUCR document* states that ECEC has multiple benefits both for individuals and society. ECEC is about “promoting participation, initiative, autonomy, problem-solving and creativity and encouraging learning dispositions to reason, investigate and collaborate” [45], and these skills are important for resilience and sustainability. The *WHO/UNICEF/Lancet Commission* shows a strong focus on the roles of families and communities as providers of care and nurturers of well-being and health for children. The document also underlines building the local community as part of a societal responsibility.

Community is a frequently used concept referring to both the community within ECEC and to the wider community and/or society. All national policy documents mention belonging/ness as a concept, though with varying emphasis. In New Zealand, the Philippines, and Slovenia, there is an affective dimension to the concept and a relational aspect that advocates care, respect, and reciprocate for others. For instance, the Philippines’ curriculum highlights the importance of the early years as the stage where “the child’s first notions of respect, acceptance, confidence, and love are developed” [41]. Both the Finnish and Swedish curricula emphasise agency and construct children as active participants in the wider society. Here, the word participation is more frequent, which s in line with the focus on citizenship.

### 3.3. Relationships between the Child and the Sociocultural and Natural Environments

In this section we describe the relationships between children and the sociocultural and natural environments in which they are embedded.

#### 3.3.1. Relationships with the Sociocultural Environment

The reviewed documents attribute a significant role to culture and environment in shaping children’s development of a resilient disposition, and ECEC is the social and cultural environment that children routinely experience. Here, we consider culture and environment in their broadest sense. Hence, the notion of environment includes each place’s geographical, physical, social, and cultural characteristics, while culture includes languages, beliefs, values, customs, traditions, and faith-based practices. This section reports our examination of policies with a focus on the relationship between the sociocultural environment and the development of resilience.

At the international level, the *Tashkent Declaration* and the *WHO-UNICEF-Lancet Commission* both highlight the importance of safe and secure environments and nurturing, caring, and responsive relationships with caregivers in young children’s holistic development, including character and disposition building. The *ARNEC document* reports a marginalisation of children’s voices and their specific needs in global and national climate change documents and notes that there are minimal environmental concerns in ECEC policies. Global challenges, such as conflicts, climate change, biodiversity loss, and natural disasters, are similarly highlighted in the *Tashkent Declaration*, which states that “all [ECEC] programmes should help children and their families to build resilience to navigate crises” [8]. The *EUCR document* brings up the importance of close collaboration and inter-agency with all services working for children, including social and health services, schools, and local stakeholders.

An environment that respects diversity and includes different cultural practices is vital for children’s development of a positive sense of belonging and self-worth. These aspects are critical in supporting resilience. The *Tashkent Declaration* points out that ECCE environments should be inclusive and equitable to support vulnerable and marginalised children and pay “attention to mother tongue language of instruction” [8]. The Slovenian, Swedish, Finnish, and New Zealand documents aspire to create non-discriminatory ECEC environments that embrace and sustain diversity, particularly cultural diversity. For example, the Swedish curriculum highlights that “no child in the preschool should be subjected to discrimination on the grounds of the gender, transgender identity or expression, ethnic origin, religion or other belief, disability, sexual orientation or age” [43]. It expects ECEC to promote children’s understanding of the value of diversity. In New Zealand, *He Māpuna te Tamaiti* suggests using responsive pedagogical practices to build caring, respectful, and reciprocal relationships with children and families. This can help children to cope with the big and small challenges they meet. It specifically emphasises the importance of fostering tuakana-teina (an indigenous Māori worldview) relationships, which means “supporting relationships within which an older or more experienced child plays with and supports a younger or less experienced child. For Māori whanau/families, taukana-teina relationships are a fundamental cultural expectation and strength” [39].

#### 3.3.2. Relationships with the Natural Environment

In addition to emphasising building human–human relationships, children’s relationships with the natural environment are similarly highlighted in the reviewed documents. Caring principles are pronounced in national documents, such as getting to know and understand the natural environment and relationships between humans and nature. Preservation and care for nature and developing appreciation, respect, and a responsible attitude towards living and non-living elements of nature are emphasised. As an example, standards about understanding the physical and natural environment appear in the Philippines’ curriculum, which promotes building children’s ability to take part in problem-solving, adapt to weather changes, and “identify cause-and-effect relationships with regards to the environment and the weather” [40]. The curriculum encourages children to socially engage and care for and maintain the environment [40].

Caring for the environment is also promoted in the Swedish, Slovenian, Finnish, and New Zealand documents. To illustrate, the Swedish curriculum states that ECEC should support children to develop responsibility for and interest in sustainable development, as well as an understanding of relations in nature, and how people, nature, and society affect each other. Similarly, the Slovenian curriculum encourages children to take action to interact, understand, respect, appreciate, and actively preserve the natural environment. In New Zealand, *He Māpuna te Tamaiti* [39] expects Kaiako (the teacher) to use resources that reflect the local environment and communities and to “draw on local knowledge to enact place-based learning” [39]. All documents suggest using a strength-based and positively focused environment to encourage children to exercise their agency in facing challenges and changes and to work through problems, thereby building resilience.

Both the *Tashkent Declaration* and the *WHO/UNICEF/Lancet Commission* recognise that the increasing severity and frequency of conflicts, emergencies, and crises weaken communities and families, threaten the well-being of young children, and risk limiting ECEC provision and quality. Whilst acknowledging that children of different ages will engage with and be impacted by climate change and environmental degradation in diverse ways, the *ARNEC document* links ecological diversity with differentiated actions that engage young children, families, and communities. It emphasises that variations between countries create different opportunities for children, families, communities, and ECEC to engage in science projects, advocacy, mitigation, and preparation in order to build resilience. The *EUCR document* does not address these issues but strongly promotes the importance of continued development of early years’ curricula to follow children’s interests, nurture their well-being, and meet the unique needs and potential of each individual child.

All in all, both national and international documents reflect an anthropocentric worldview where the divide between humans and the more-than-human world is not problematised or bridged.

## 4. Discussion

The aim of this study was to explore how ECEC might contribute to resilience in a world of change. Based on five national and four international documents, our analysis showed that resilience is addressed, although mostly implicitly.

Resilience is predominantly described as a capacity within the individual child. There is a strong focus on psychological resilience, in terms of supporting the emotional and social skills and agency of children. This is in line with Masten and Barnes [6], who mention agency as a common factor in resilience. It also resonates with childism, which foregrounds the active and competent child [27]. From an ECEfS perspective, ECEC can facilitate a strong foundation for children to become active citizens who contribute to local and global communities, thus strengthening their agency in the transformation towards a more sustainable world. The concept of ´citizen scientist´ acknowledges the child as a solution-oriented, creative actor in change.

In some of the analysed documents, the importance of community and close relationships is revealed as a facilitator of resilience. The documents mainly describe how the community can support the resilience of the child, but we argue that in alignment with contemporary theory on resilience, there is a need to emphasise that resilience is a shared and relational capacity [6] and to focus on the reciprocity of relationships between children, their families, and communities [7]. As resilience is a shared capacity within the community, we need to acknowledge the entanglement of children and their cultural and natural environments as well as the importance of strengthening the experience of this interconnectedness. From this perspective, place-based theory, as described by Grunewald [29], can offer an in-depth understanding of the development of resilience and how it can be promoted within the ECEC context.

Education plays a crucial role in influencing the ecological well-being of places [29,31]. This article applied the twin processes of cultural decolonisation and ecological rehabilitation in the critical pedagogy of place approach [29,31], thus advocating for increased attention to the development of culturally inclusive and responsive policies and pedagogies. Teachers, children, and families should be encouraged to decolonise and unlearn dominant and mainstream practices that damage human–human and human–more-than-human relationships, thereby restoring the ecological well-being of all inhabitants. The reviewed national policies consider respecting and including diverse cultural practices and languages pivotal to supporting children’s development of a sense of belonging, self-worth, and resilience. Inclusive practices that embrace diversity sustain diverse cultures and, more importantly, prevent biodiversity loss in the natural environment because they model caring and relational practices. Appreciation for diversity—in its broadest sense—is vital to embed in regional and global policies so that recommendations reflect and are applicable to the diverse contexts and lived realities of young children, their families, and communities. Creating place-based experiences makes it possible for children to understand the ecology of the places they inhabit and learn how to share and live their lives with humans and more-than-human inhabitants [29,31]. Further, multisystem perspectives on resilience [7] can contribute to building resilience that supports communities working collectively and interconnectedly within their contexts.

The study reveals that national and international frameworks generally address diversity in terms of gender, age, language, and to some extent socioeconomic background. Although all of the national policies promote non-discriminatory ECEC pedagogies, they are not representative and reflective of indigenous cultures, with New Zealand as the only exception. Considering the fact that the Sámi (Europe´s only indigenous people) live in both Sweden and Finland, we argue that this omission is problematic and suggest that more attention should be given to the heterogeneity and inclusion of multiple voices. In Chan and Ritchie [46], the authors explain how indigenous Māori knowledge respects and values, rather than exploits, what the natural environment offers and profits from it. These authors suggest using a critical pedagogy of place [29,31] to inform inclusive and equitable practices in ECEC settings. Further, indigenous knowledge can contribute to the transformation towards more relational worldviews, showing how to create a balance between humans and the more-than-human world. In line with current research in ECEfS, we argue that indigenous perspectives must be embraced within ECEC.

New Zealand policies also embrace using local knowledge to strengthen children’s sense of belonging in communities or places in which they learn and live. An understanding of and connection to local places enable children and their families to construct social networks for community members in order to collectively solve problems and crises and repair and rebuild human–human and human–environment relationships, thereby relearning how to care for and share places [29,31]. Nonetheless, we want to point out that although these national policies promote inclusive and non-discriminatory ECEC pedagogies, the *ARNEC document* reports an exclusion of children’s voices in climate and environmental discussions and the *EUCR document* does not mention these issues at all. We argue that adults are role models for children. They must demonstrate inclusive practices to show children how to prevent possible cultural colonisation and dominance, and instead establish caring, responsive, and equitable relationships [29,30].

It is clear from the reviewed documents that there is a significant gap between national and international policies regarding the interrelated notions of resilience and sustainability. The role of ECEC in supporting children and families to build resilience in order to navigate environmental crises and sustain healthy and habitable planetary ecosystems was recently highlighted in the *Tashkent Declaration,* the *WHO/UNICEF/Lancet Commission,* and the *ARNEC document*. Nevertheless, this review of national policies suggests that further work must be done to meet these expectations. Natural disasters are becoming increasingly common, frequent, and catastrophic. We argue that resilience may run out without multisystem collective efforts.

All in all, we argue that national as well as international policies still reflect an anthropocentric worldview, which neither supports the resilience of children nor the rapid transformation of our societies towards sustainable ways of living. Hence, the fact that resilience can be strengthened by realising the entanglement of humans and the more-than-human world is neglected, which is in stark contrast with previous research on the importance of nature contact and nature connectedness for children´s health, well-being, and resilience [13,15,21].

We argue that policies should be developed and contextualised with an emphasis on the interconnectedness of humans and the more-than-human world. In alignment with the Common Worlds Research Collective [47], we suggest that education should not be about the world but with the world in which humans are inextricably entangled and embedded. Particularly, there is a potential for ECEC to acknowledge the collective and relational agency of human and non-human actors, foster the interdependence and interconnectedness of these actors, and support ecological justice. For now, there are few expressions pointing to the dynamic and relational understanding of resilience that is relevant for children to meet the challenges of the planetary emergency.

## 5. Conclusions

By promoting the concept of resilience, ECEC has the potential to support children, communities, and societies in meeting the challenges of a world in rapid change, and it facilitates the urgent transformation towards a sustainable world. Explicitly including the concept in ECEC policies would put a spotlight on direct goals and practical strategies that support the health, well-being, and agency of children, thereby empowering them to be part of transforming the communities and societies in which they live. Resilience must not be understood as the capacity of a single individual to persist and respond to the sustainability crisis, but rather, it is imperative to acknowledge these entanglements within the community (human and more-than-human) and promote necessary structural changes. Hence, we advocate an understanding of the concept of resilience as a relational, dynamic, and holistic capacity encompassing physical, psychological, and social dimensions. ECEC would benefit from integrating multisystemic perspectives of resilience by considering and including disciplinary knowledge from natural and social sciences. We suggest using resilience-oriented pedagogies in ECEC to foster a more sustainable future for both people and the planet.

## 6. Limitations and Future Research

Whilst this study has examined the national documents of five countries, it does not capture the “voices” of African and Pacific nations, which have experienced severe draught, flooding, and irreversible environmental damage in recent decades. Additionally, the policies and documents reflect commitments and aspirations only. How they are enacted in ECEC settings and changes in action are equally important. An exploration of how ECEC settings promote resilience and sustainability is beyond the scope of this study but a valuable future research project.

## Data Availability

All documents analysed appear in the references.
